# RETRACTION: Effect of Intramedullary Nail Fixation and Internal Plate Fixation in Distal Tibia Fracture Surgery on Post‐Operative Wound Infection in Patients: A Meta‐Analysis

**DOI:** 10.1111/iwj.70348

**Published:** 2025-03-17

## Abstract

**RETRACTION:** LvC. , JiangC. , LvW. , ZhangS. , and LiC. , “Effect of Intramedullary Nail Fixation and Internal Plate Fixation in Distal Tibia Fracture Surgery on Post‐Operative Wound Infection in Patients: A Meta‐Analysis,” International Wound Journal 21, no. 2 (2024): e14383, 10.1111/iwj.14383.PMC1082812637828714

The above article, published online on 12 October 2023, in Wiley Online Library (http://onlinelibrary.wiley.com/), has been retracted by agreement between the journal Editor in Chief, Professor Keith Harding; and John Wiley & Sons Ltd. Following an investigation by the publisher, all parties have concluded that this article was accepted solely on the basis of a compromised peer review process. The editors have therefore decided to retract the article. The authors did not respond to our notice regarding the retraction.

**RETRACTION:** C. Lv , C. Jiang , W. Lv , S. Zhang , and C. Li , “Effect of Intramedullary Nail Fixation and Internal Plate Fixation in Distal Tibia Fracture Surgery on Post‐Operative Wound Infection in Patients: A Meta‐Analysis,” International Wound Journal 21, no. 2 (2024): e14383, 10.1111/iwj.14383.PMC1082812637828714

The above article, published online on 12 October 2023, in Wiley Online Library (http://onlinelibrary.wiley.com/), has been retracted by agreement between the journal Editor in Chief, Professor Keith Harding; and John Wiley & Sons Ltd. Following an investigation by the publisher, all parties have concluded that this article was accepted solely on the basis of a compromised peer review process. The editors have therefore decided to retract the article. The authors did not respond to our notice regarding the retraction.

